# Ca^2+^ Signaling Occurs via Second Messenger Release from Intraorganelle Synthesis Sites

**DOI:** 10.1016/j.cub.2008.09.024

**Published:** 2008-10-28

**Authors:** Lianne C. Davis, Anthony J. Morgan, Margarida Ruas, Julian L. Wong, Richard M. Graeff, Albert J. Poustka, Hon Cheung Lee, Gary M. Wessel, John Parrington, Antony Galione

**Affiliations:** 1 Department of Pharmacology, University of Oxford, Mansfield Road, Oxford OX1 3QT, United Kingdom; 2 Department of Molecular Biology, Cellular Biology and Biochemistry, Brown University, 185 Meeting Street, Box G-L173, Providence, Rhode Island 02912; 3 Department of Pharmacology, University of Minnesota, 4-145 Jackson Hall, 321 Church St SE, Minneapolis, Minnesota 55455; 4 Evolution and Development Group, Max-Planck Institute Für Molekulare Genetik, Ihnestrasse 73, 14195 Berlin, Germany; 5 Department of Physiology, University of Hong Kong, 4/F Lab Block, Faculty of Medicine Building, 21 Sassoon Road, Hong Kong, People's Republic of China

**Keywords:** SIGNALING

## Abstract

Cyclic ADP-ribose is an important Ca^2+^-mobilizing cytosolic messenger synthesized from β-NAD^+^ by ADP-ribosyl cyclases (ARCs). However, the focus upon ectocellular mammalian ARCs (CD38 and CD157) has led to confusion as to how extracellular enzymes generate intracellular messengers in response to stimuli. We have cloned and characterized three ARCs in the sea urchin egg and found that endogenous ARCβ and ARCγ are intracellular and located within the lumen of acidic, exocytotic vesicles, where they are optimally active. Intraorganelle ARCs are shielded from cytosolic substrate and targets by the organelle membrane, but this barrier is circumvented by nucleotide transport. We show that a β-NAD^+^ transporter provides ARC substrate that is converted luminally to cADPR, which, in turn, is shuttled out to the cytosol via a separate cADPR transporter. Moreover, nucleotide transport is integral to ARC activity physiologically because three transport inhibitors all inhibited the fertilization-induced Ca^2+^ wave that is dependent upon cADPR. This represents a novel signaling mechanism whereby an extracellular stimulus increases the concentration of a second messenger by promoting messenger transport from intraorganelle synthesis sites to the cytosol.

## Results and Discussion

The mechanisms coupling extracellular stimuli to synthesis of the Ca^2+^-mobilizing messenger cyclic ADP-ribose (cADPR) are poorly understood. ADP-ribosyl cyclases (ARCs) synthesize cADPR from β-NAD^+^ [1, 2] (and possibly another Ca^2+^-mobilizing messenger, NAADP, though this remains uncertain physiologically [3, 4]). The fact that mammalian ARC isoforms (CD38 and CD157) appear to be primarily ectocellular [1, 2] has raised questions as to how they could generate intracellular messengers in a regulated manner [5].

### Cloning of Sea Urchin ARCs

To address these issues, we cloned and characterized three ARC enzymes from sea urchin egg where cADPR and ARC were first discovered [6]. Using CD38, CD157, and *Aplysia* ARC as query sequences to probe the *Strongylocentrotus purpuratus* genome [7] and expressed-sequence tag (EST) cDNA databases [8], we identified three ARC isoforms, ARCα, ARCβ, and ARCγ. To obtain the complete cDNA sequences, we designed gene-specific primers and carried out RT-PCR with total RNA from *S. purpuratus* eggs, ovaries, or testes. The cDNA sequences obtained for ARCα, ARCβ, and ARCγ have open reading frames corresponding to proteins with predicted molecular masses of 36.3, 35.4, and 40.1 kDa, all containing the ARC domain (Rib-hydrolase), catalytic residues, and critical disulphide bridges (Figure S1A available online) [1]. The mRNAs for all three ARCs were found in ovary, testis, and egg (Figure S1D). Recently, sea urchin ARCs were cloned via a different approach [9], and our slightly different sequences are probably attributable to polymorphisms [7] identified in multiple independent RT-PCR clones (Figure S1E). Sea urchin ARCs are predicted to be membrane bound with different modes of membrane attachment (Figure S1F).

### Immunolocalization of ARCs in Sea Urchin Eggs

**Figure 1:**
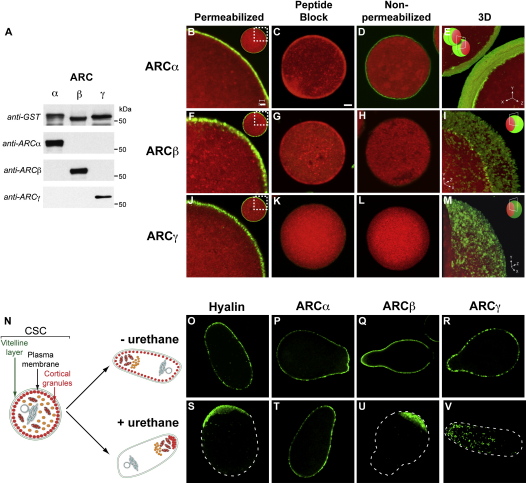
Distribution of ARCα, ARCβ, and ARCγ in Sea Urchin Eggs (A) Specificity of ARC antibodies confirmed in immunoblots with recombinant GST-ARCs. (B–M) Eggs stained with Lysotracker Red DND-99 (red) were fixed, permeabilized (unless otherwise indicated), and labeled (green) with antibodies against ARCα (B–E), ARCβ (F–I), and ARCγ (J–M). The following are shown: cortical staining with ARC antibodies (B, F, and J); staining blocked with competing antigenic peptides (C, G, and K); nonpermeabilized eggs (D, H, and L); and 3D reconstruction of sequential z sections (E, I, and M). (N–V) Stratified eggs studies. (N) Schematic representation of the stratification of intracellular organelles. Staining for the cortical granule marker protein, hyalin, or ARCs in the absence (O–R) or presence (S–V) of urethane is shown. Scale bars represent 2 μm (B) and 10 μm (C).

The subcellular distribution of endogenous ARCs is currently unknown, so we first examined immunofluorescence in eggs costained with Lysotracker Red to label acidic organelles. In fixed, permeabilized eggs, specific ARC staining was peripheral for all isoforms (Figures 1B, 1F, and 1J) but with crucial differences: ARCα staining was ectocellular at the outer edge of the Lysotracker Red staining (Figure 1B), was smooth in three-dimensional reconstructions (Figure 1E), and was even observed in nonpermeabilized eggs (Figure 1D). In contrast, ARCβ and ARCγ were intracellular proteins that colocalized with Lysotracker Red (Figures 1F and 1J), distributed in punctate vesicles 1–2 μm in diameter (Figures 1I and 1M), and not observed in nonpermeabilized eggs (Figures 1H and 1L). The distribution of ARCβ and ARCγ was not mimicked by endoplasmic reticulum (ER) staining (Figure S2).

Intriguingly, the localization of ARCβ and ARCγ was reminiscent of cortical granules, the acidic, exocytotic vesicles that are docked at the plasma membrane (Figure 1N) to generate the fertilization envelope [10]. We confirmed this locus in a number of ways. First, unlike other organelles, cortical granules do not migrate upon stratification of live eggs by centrifugation unless pretreated with urethane (Figures 1O and 1S) [11]. Accordingly, ARCβ and ARCγ remained peripheral in untreated stratified eggs (Figures 1Q and 1R), but were dislodged and migrated in urethane-treated eggs (Figures 1U and 1V). In contrast, ARCα staining remained peripheral with (Figure 1T) or without (Figure 1P) urethane, as expected for a plasma membrane and/or vitelline layer (PMVL, Figure 1N) protein.

**Figure 2:**
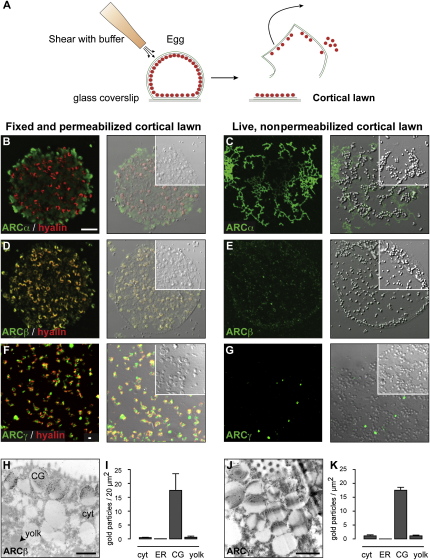
ARCβ and ARCγ Localize to the Cortical Granule Lumen Schematic representation (A) of cortical lawn preparation by the shearing of eggs adhering to coverslips (B–G). Cortical lawns stained (green) for ARCα (B and C), ARCβ (D and E), ARCγ (F and G) in fixed, permeabilized (B, D, and F), or live, unpermeabilized samples (C, E, and G). Fixed samples were costained for the cortical granule protein hyalin (red). Fluorescence images are shown (left) and are overlayed with DIC images (right). (H–K) Immunogold localization and quantification of ARCβ (H and I) and ARCγ (J and K). For clarity, micrographs represent magnified cortical regions that do not include the entire region used for quantification. (I and K) Quantification of labeling in cytosol (cyt), endoplasmic reticulum (ER), cortical granules (CG), and yolk platelets (yolk); vertical bars display standard deviation for tallies from six different eggs. The images in (B)–(E) are depicted on a common scale; those in (F) and (G) share a different scale. Scale bars represents 10 μm (B) and 1 μm (F, H, and J).

Second, by the shearing of adherent eggs (Figure 2A), cortical lawns that comprised cortical ER, docked cortical granules, and PMVL were prepared [12]. Spheroid cortical granules stained positively for hyalin (Figures 2B, 2D, and 2F), which colocalized with ARCβ and ARCγ (Figures 2D and 2F) but not ARCα (Figures 2B and 2C). Importantly, ARCβ and ARCγ appeared to be luminal because immunostaining was not seen in unpermeabilized lawns (Figures 2E and 2G).

Third, electron microscopy and immunogold labeling confirmed that ARCβ and ARCγ associate with the cortical granule lumen, but not with underlying yolk platelets, cytosol, or ER (Figures 2H–2K). The data support an ectocellular ARCα, whereas ARCβ and ARCγ are within the cortical granule lumen.

### pH Dependence of ARC Activity and Distribution in Subcellular Fractions

**Figure 3:**
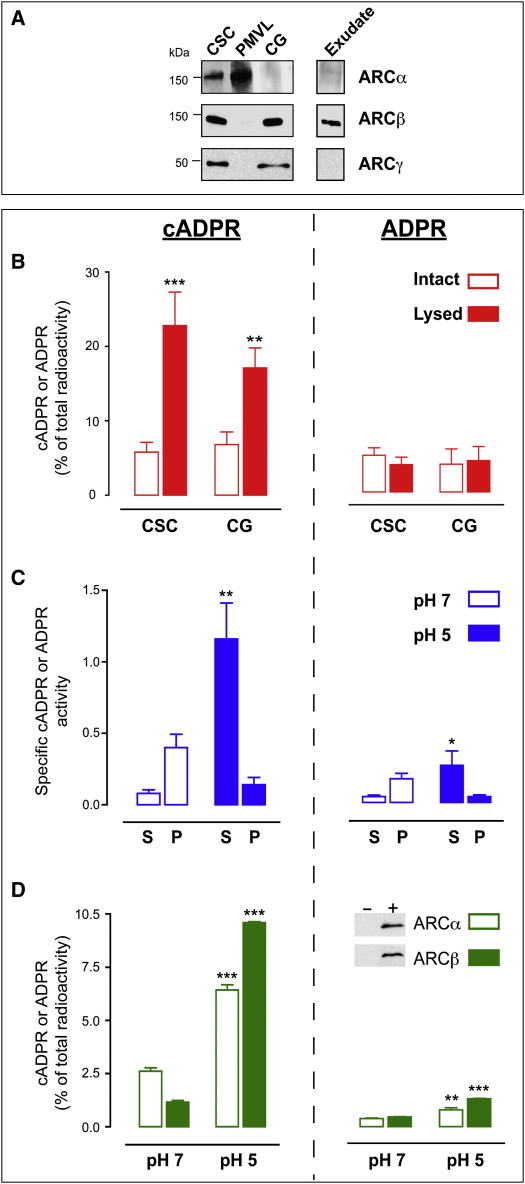
ARC Distribution and Activity in Egg Subcellular Fractions (A) Immunoblotting of egg fractions for ARCα, ARCβ, and ARCγ. ARC isoforms are present as tetramers or monomers, as seen with other ARCs [38, 39]. (B–D) ARC activity assessed by [^32^P]cADPR and [^32^P]ADPR production. (B) CSCs and CGs were resuspended in either isotonic (Intact) or hypotonic buffers (Lysis). (C) CGs were lysed in hypotonic buffer at pH 7 or pH 5 and separated into soluble (S) and particulate (P) fractions. (D) pH dependency of recombinant ARCα or ARCβ is shown. Inset shows western blots before (−) and after (+) induction in yeast by methanol. ^∗∗^, p < 0.01; ^∗∗∗^, p < 0.001 (n = 3). Data are presented as the mean ± SEM of n populations.

We extended these studies by using biochemical cell fractionation. Cell-surface complexes (CSCs, Figure 1N) were prepared and further fractionated into their constituent PMVL and cortical granules where indicated. In agreement with the above, ARCα was only detected in CSCs and PMVL (Figure 3A), whereas ARCβ and ARCγ were confirmed in CSCs and cortical granules (Figure 3A).

A luminal localization of ARCβ was further reinforced. First, eggs were treated with Ca^2+^ ionophore to induce cortical granule exocytosis, and ARCβ was then detected in the surrounding exudate (Figure 3A), consistent with its release from the cortical granule lumen, possibly by clipping of the putative transmembrane domain (Figure S1F) by resident proteases [10]. By contrast, ARCα and ARCγ did not appear in the exudate (Figure 3A), presumably because of membrane attachment (Figure S1F). Second, ARC activity in CSCs or cortical granules was enhanced upon vesicle lysis, consistent with restricted substrate access when intact (Figure 3B). Taken together, these data overwhelmingly suggest that ARCα is ectocellular whereas ARCβ and ARCγ reside within the lumen of the cortical granule, the first molecularly identified ARCs to be found exclusively within an organelle.

Our conclusion differs with the heterologous expression of one recombinant ARC (equivalent to our ARCβ) localized in the ER [9]. One possibility is that this is a function of the expression system because another cortical granule protein (SFE9) is also retained within the ER when expressed heterologously (data not shown).

We next asked whether luminal ARCs are active. Cortical granules are acidic vesicles (pH ∼5.5, [10]), and, fittingly, recombinant (Figure 3D) and endogenous (Figure 3C) sea urchin ARCs preferentially synthesize cADPR (and not ADPR) at acidic pH. Lysed cortical granules were also separated into membranes, and soluble proteins and ARC activity was greater for the soluble fraction at pH 5 (Figure 3C). The low activity in the particulate fraction at pH 5 (close to the predicted pIs of 4.7 and 5.7 for ARCβ and ARCγ, respectively) is probably due to protein aggregation and precipitation at this pH (data not shown; cf. CD38 inactivation [13]). Our findings confirm that ARC activity is compartmentalized within the lumen of an organelle with an optimal pH.

Exoplasmic sea urchin ARCs contrast with reports of soluble ARC activity [14, 15], but this may be an artifact of homogenate preparation where Ca^2+^ contamination promotes cortical granule exocytosis [12] and the disgorging of ARC to the “cytosol.” Homogenate prepared in the presence of EGTA [glycol-bis(2-aminoethylether)-tetra acetic acid] preserves vesicular integrity and ARC remains luminal (Figure S3) until lysed or treated with excess Ca^2+^ (Figure S3).

### Nucleotide Transport and Fertilization Ca^2+^ Signals

If ARCs are luminally active, then substrates and products must be transported in and out during stimulation (Figure S4) and the small ARC activity in intact CSCs and cortical granules is consistent with transport (Figure 3B). Therefore, we investigated the effect of transport inhibitors upon physiological Ca^2+^ responses.

**Figure 4:**
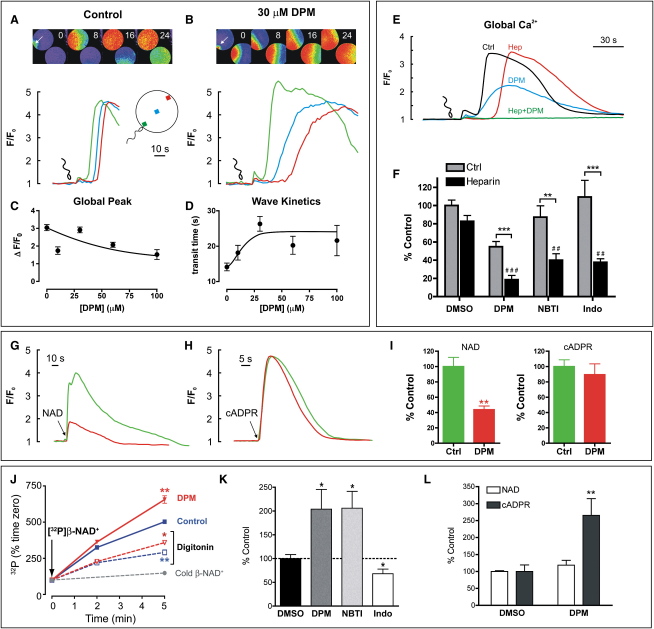
Nucleotide Transport in Cortical Granules Is Required for Fertilization Ca^2+^ Responses (A–D) Effect of dipyridamole (DPM) upon fertilization-induced Ca^2+^ signals. Ca^2+^ waves in control (plus DMSO) (A) and DPM-treated eggs (B) are shown; image numbers represent the time (s) after wave initiation. The intracellular [DPM] is ∼10% of the extracellular concentration (Figure S5). The effect of DPM upon the global main Ca^2+^ spike (C) and wave kinetics (D) is shown (n = 4–32 eggs). (E and F) Effect of transport inhibitors with or without heparin (250 mg/ml pipette concentration) upon fertilization-evoked Ca^2+^ signals. (E) Traces represent global Ca^2+^ signals upon fertilization in control (Ctrl) and heparin-injected (hep) eggs incubated with either DMSO or 30 μM DPM. (F) A summary of the effect of DPM, indoprofen (indo), and nitrobenzylthioinosine (NBTI) upon the main Ca^2+^ spike amplitude in eggs injected with or without heparin is shown (n = 12–32). Heparin increased the lag from 13 ± 1 s to 38 ± 2 s (n = 29–33, p < 0.001). Responses are normalized to the ΔF/F_0_ in control eggs (ΔF/F_0_ = 2.38 ± 0.19, n = 32). Eggs were treated with 30 μM DPM for 3 min, 500 μM indoprofen for 5 min, or 100 μM NBTI for 45–90 min (^∗∗^, p < 0.01 and ^∗∗∗^, p < 0.001, comparing inhibitors with or without heparin; ##, p < 0.01 and ###, p < 0.001 versus heparin plus DMSO). (G–I) Effect of DPM upon signals in response to nucleotide injection: 30 μM DPM inhibits Ca^2+^ responses to microinjection of β-NAD^+^ (5–10 mM pipette concentration [G and I]) but not to cADPR (30 μM pipette concentration [H and I]); n = 44–51 eggs. (G and H) Control ΔF/F_0_: β-NAD^+^, 2.71 ± 0.31 (n = 44); cADPR, 2.19 ± 0.19 (n = 50); without nucleotides, 0.43 ± 0.10 (n = 13). I: ΔF/F_0_ expressed as percentage of control. (J) Kinetics of the accumulation of ^32^P in CSCs incubated with [^32^P]β-NAD^+^ in the presence of DMSO vehicle (blue) or 10 μM DPM (red). Digitonin (300 μM) and 100 μM unlabeled (“cold”) β-NAD^+^ were added where indicated (n = 3). ^∗^, p < 0.05 and ^∗∗^, p < 0.01 versus control (radioactivity at time zero = 1947 ± 116 disintegrations per minute [d.p.m.]). (K) Net luminal ^32^P accumulation (Intact minus digitonin) after a 5 min incubation of CSC with [^32^P]β-NAD^+^ in the presence of 0.5% DMSO, 10 μM DPM, 10 μM NBTI, or 100 μM indoprofen (n = 5–7, ^∗^p < 0.05 versus DMSO). Raw radioactivity of DMSO control, 3251 ± 658 d.p.m. (L) Identification of luminal ^32^P-labeled nucleotides by TLC analysis, after an 18 min incubation of CSC with [^32^P]β-NAD^+^ in the presence of 0.8% DMSO or 16 μM DPM (n = 4, ^∗∗^p < 0.01 versus DMSO). Spot intensities (arbitrary units) with DMSO are as follows: β-NAD^+^, 1696 ± 274; cADPR, 354 ± 94. Note that in these experiments, [^32^P]ADPR levels were not consistently above the level of detection. Data are presented as the mean ± SEM of n populations.

At fertilization, cADPR contributes to the prominent Ca^2+^ wave (spike upstroke) [16, 17] via ARC [18, 19]. In control eggs, fertilization induced a cortical flash followed by a Ca^2+^ wave that crossed the egg in ∼14 s (Figures 4A and 4D). With the cADPR transport inhibitor dipyridamole (DPM) [5, 20], the early events (the cortical flash and Ca^2+^ wave initiation) were relatively unaffected (Figures S6A and S6B; p > 0.05), whereas the wave transit time (p < 0.01, Figures 4B and 4D) and the antipodal Ca^2+^ release (p < 0.01, Figure 4B, Figure S6B) were dramatically slowed. Globally, the Ca^2+^ spike amplitude was reduced (p < 0.01, Figures 4C and 4F). Note that the intracellular DPM concentration is ∼10% of the extracellular (Figure S5). This partial inhibition by DPM mimics cADPR antagonism or ryanodine receptor inhibition [16, 17].

At fertilization, cADPR acts synergistically with IP_3_, and, consequently, cADPR antagonism appears more striking when IP_3_ is antagonized with heparin [16, 17]. Heparin alone did not affect the Ca^2+^ spike amplitude but extended the lag (p > 0.05, Figures 4E and 4F). However, heparin and DPM together almost completely blocked the Ca^2+^ response (Figures 4E and 4F), consistent with DPM acting upon the cADPR pathway.

We also tested other nucleotide transport inhibitors, nitrobenzylthioinosine (NBTI) [5, 20] and indoprofen [21, 22]. Alone, these agents had no effect on the Ca^2+^ spike amplitude (Figure 4F, p > 0.05), but, like DPM, they synergistically inhibited the response in heparinized eggs (albeit less efficaciously than DPM, Figure 4F). Note that at relevant concentrations, these inhibitors did not directly inhibit Ca^2+^ release evoked by IP_3_, cADPR, or NAADP (Figures S7A–S7C), nor did they directly inhibit ARC (Figure S7D). All inhibitors act in a manner diagnostic for antagonism of the cADPR pathway.

DPM was used to narrow down the locus of action. We circumvented fertilization by injecting ARC substrate, β-NAD^+^, which releases Ca^2+^ upon its conversion to cADPR [18, 19]. DPM blocked the Ca^2+^ response to β-NAD^+^ (p < 0.01) but not to the product, cADPR (p > 0.05, Figures 4G–4I), pointing to an inhibition of ARC that must be indirect (Figure S7D). We also discount side effects of transport inhibitors upon Ca^2+^ removal (Figure 4H), and upon the activity of cyclic nucleotide phosphodiesterases and cyclo-oxygenase (Figure S8).

More is known about plasmalemmal β-NAD^+^ and cADPR transport [5, 20, 23, 24] than organelle nucleotide fluxes [25–27]. Therefore, we characterized transport in cortical granules by using [^32^P]β-NAD^+^ (Figures 4J–4L). [^32^P]β-NAD^+^ uptake into the lumen via specific sites was confirmed with digitonin lysis and unlabeled β-NAD^+^, respectively. DPM enhanced ^32^P accumulation (p < 0.001, Figure 4J) in a dose-dependent manner (Figure S9) and was replicated by NBTI at 1 μM (175 ± 15%, n = 7) and 10 μM (Figure 4K). By contrast, indoprofen reduced ^32^P accumulation (Figure 4K).

Our explanation is that indoprofen affects the β-NAD^+^ transporter, whereas DPM and NBTI block [^32^P]cADPR efflux [5], thereby trapping it in the lumen. Indeed, thin-layer chromatography (TLC) analysis confirmed that [^32^P]cADPR is generated within the lumen and that DPM selectively enhanced [^32^P]cADPR accumulation (Figure 4L). We conclude that organellar nucleotide transport is an integral element of the ARC signaling cascade.

### Conclusions

Our understanding of ARC regulation has been hitherto confounded by the focus upon ectocellular ARCs [5]. Instead, we propose that ARCs natively sequestered within acidic organelles are the most important forms. Reports of ARCs that are intracellular [2, 5, 28] and capable of synthesizing cADPR at acidic pH [29–31] hint at a significance beyond the sea urchin egg. At fertilization, ARCβ and ARCγ locally provide cADPR for cortical Ca^2+^ signals to drive exocytosis [16, 32] and then for global Ca^2+^ waves following cADPR diffusion [32]. We speculate that multiple mechanisms modulate ARC at fertilization (Figure S4), including transporter phosphorylation (possibly via cGMP [15, 18]), changes in cortical granule pH [10, 33], and exocytosis. Subsequent endocytosis (and endosomal acidification) [5, 34] could even replenish and reactivate membrane-bound ARCs.

In spite of the presence of ARCα, ectocellular synthesis of cADPR cannot occur [35], probably because of the inhibitory pH of sea water. However, ectocellular proteins reach the cell surface via the lumen of a secretory pathway [36], and ARCα is indeed vesicular in immature oocytes (Figure S10), where it may play a role during egg maturation when cADPR responses begin to appear [37]. Thus ARC effectiveness may depend upon when it is in its active, vesicular form.

The identity of the transporters is unclear, but the differential pharmacology of β-NAD^+^ uptake and cADPR release suggests different proteins. Nonetheless, we can exclude the candidate β-NAD^+^ transporter, connexin 43 [5, 24], because connexins are absent from the sea urchin genome [7].

In summary, we propose a model whereby cell-surface stimuli couple to vesicular ARCs by regulating vesicle substrate and/or product transporters and vesicle pH. Our results further raise the profile of acidic vesicles as Ca^2+^-signaling organelles—not only are they Ca^2+^ stores [33], but they are also reservoirs of second messenger mobilized by physiological stimuli.

## Experimental Procedures

### Cloning of *S. purpuratus* ARC cDNA Sequences

Full-length cDNAs were generated by RT-PCR from total RNA with the use of specific primers and the SuperScriptII One-Step RT-PCR system with Platinum *Taq* High Fidelity and cloned into the pCRII-TOPO vector (Invitrogen).

### Production of Recombinant ARC Proteins

Rib-hydrolase domains of ARCα, ARCβ, and ARCγ were cloned into pGEX-2TKP and pPICZαA for expression either in bacteria (as GST-fusion proteins) or in yeast (as secreted proteins with mutated N-glycosylation sites), respectively.

### Preparations of Eggs and Subfractions

For stratification, dejellied eggs were centrifuged on a cushion of 1 M sucrose at 17,500 × g for 30 min. For dislodging cortical granules, eggs were suspended in 400 mM urethane immediately before and during centrifugation. Published methods were used to prepare cortical lawns, egg homogenates, CSCs, and cortical granules (CGs) (Supplemental Data). Exudates were collected from A23187-stimulated live eggs.

CSCs or CGs were hypotonically lysed in 10 mM HEPES (pH 7) or 10 mM acetate (pH 5), as confirmed by the release of Lysotracker Red (data not shown). The particulate and soluble fractions were separated by ultracentrifugation at 100,000 × g for 1 hr.

### Immunolocalization

Antibodies specific for ARC isoforms were raised commercially in rabbits with specific peptides (ARCα and ARCβ, Covalab; ARCγ, Eurogentec) and affinity purified. Eggs and cortical lawns were fixed with 4% paraformaldehyde and ovary sections with 10% formalin. Permeabilization was effected with 0.2% Triton X-100 where indicated. Some egg preparations were stained with 1 μM Lysotracker Red DND-99 (Invitrogen) for 20 min prior to fixation. Primary antibody staining was visualized with fluorescently labeled secondary antibodies and confocal laser-scanning microscopy with standard green or red filters.

For electron microscopy, eggs were fixed, mounted, and visualized with affinity-purified anti-ARC antibodies and secondary antibodies conjugated to 15 nm colloidal gold particles.

### ARC Activity

Protein samples were incubated with 16 nM [^32^P]β-NAD^+^ (GE Healthcare) for 1 hr at 20°C, and [^32^P]cADPR was separated by TLC and visualized with a storage phosphor screen (Typhoon, GE Healthcare).

Alternatively, cyclization of nonradioactive β-NAD^+^ was assessed in egg homogenates (without cortical granules) by monitoring the Ca^2+^ release evoked by cADPR product with fluo-3.

### Nucleotide Uptake

CSCs were incubated with 16 nM [^32^P]β-NAD^+^ and bound and free separated by vacuum filtration and three washes (with or without 300 μM digitonin). Nonspecific binding was defined by unlabeled 100 μM β-NAD^+^. Where indicated, DPM, NBTI, or Indoprofen was preincubated for 2 min prior to addition of [^32^P]β-NAD^+^.

For identification of which ^32^P-labeled nucleotides accumulate in the cortical granule lumen, CSCs were incubated with 50 nM [^32^P]β-NAD^+^ for 18 min, filtered and washed to remove extravesicular nucleotides, and lysed in 4.8% TCA. Precipitated protein was pelleted, and supernatants were concentrated ∼10-fold under vacuum before nucleotide TLC analysis as above. Time-zero intensities were subtracted from each nucleotide spot.

### Ca^2+^ Fluxes

Ca^2+^ concentration was determined in egg homogenates in a fluorimeter with 3 μM fluo-3 (excitation 506 nm, emission 526 nm) or in intact eggs microinjected with fluo-4 dextran (1 mM in the pipette; excitation 488 nm, emission > 505 nm). Nucleotides were microinjected with an inert marker (Alexa Fluor 647 Dextran, 50 μM in the pipette; excitation 633 nm, emission > 650 nm). Pipette concentrations were as follows: β-NAD^+^ (5–10 mM, plus 100 μM EGTA) and cADPR (30 μM). To estimate the intracellular DPM concentration, we monitored the intrinsic, UV fluorescence of DPM in intact eggs (excitation 364 nm, emission > 385 nm).

### Data Analysis

Data are presented as the mean ± standard error of the mean (SEM) of n preparations, statistically analyzed with Student's t test (for two means) or an analysis of variance followed by a Tukey or Dunnett post test (for multiple means), and significance was assumed at p < 0.05.

## Accession Numbers

cDNA sequences are deposited in GenBank with accession numbers EF544483 (ARCα), EF544484 (ARCβ) and EF544485 (ARCγ).

## Supplemental Data

Document S1. Supplemental Experimental Procedures and Ten Figures
