# Supplementary material for: Ca2+ Signaling Occurs via Second Messenger Release from Intraorganelle Synthesis Sites
Source: Curr Biol. 2008 Oct 28;18(20-24):1612–8. doi: 10.1016/j.cub.2008.09.024 (PMC2581485; doi:10.1016/j.cub.2008.09.024)
Supplement: Document S1. Supplemental Experimental Procedures and Ten Figures [file mmc1.pdf]

Supplement Data

Ca^{2+} Signaling Occurs via Second Messenger Release from Intraorganelle Synthesis Sites

Lianne C. Davis, Anthony J. Morgan, Margarida Ruas, Julian L. Wong, Richard M. Graeff, Albert J. Poustka, Hon Cheung Lee, Gary M. Wessel, John Parrington, and Antony Galione

SUPPLEMENTAL EXPERIMENTAL PROCEDURES

RNA extraction

S. purpuratus ovary or testis were quickly frozen in liquid nitrogen and stored at -80ºC. RNA extraction was conducted using a RNeasy system (QIAGEN) following the manufacturer's instructions. Extracted RNA was DNase treated and purified using RNeasy MinElute Cleanup spin columns (QIAGEN). Aliquots of DNase-treated RNA were stored at -80ºC. A similar protocol was followed for shed eggs.

Cloning of *S. purpuratus* ARC cDNA sequences

cDNA sequence corresponding to the 3'-end of ARCα was obtained by 3'-RACE using a BD SMART RACE cDNA amplification kit (BD biosciences), following the manufacturer's instructions. 1 μg of *S. purpuratus* egg total RNA was used for the synthesis of first-strand cDNA and a gene specific primer based on an EST sequence (5'-CATTTGCGACCTTCTGGGATCGTGT-3') was used in the amplification of the 3'-end

of the cDNA using the BD Advantage 2 PCR Kit following the parameters: (a) 5 cycles of 94°C (30 s), 72°C (3 min); (b) 5 cycles of 94°C (30 s), 70°C (30 s), 72°C (3 min); (c) 45 cycles of 94°C (30 s), 65°C (30 s) and 72°C (10 min).

Full-length cDNAs were generated by RT-PCR from total RNA using the SuperScript II One-Step RT-PCR system with Platinum *Taq* High Fidelity (Invitrogen) and gene specific primers for the three isoforms:

<u>ARCα</u>

For: 5'-ATGATGAATCTCCTCCGCACG-3'

Rev: 5'-CGAATTATGCTTCTTGAG-3'

<u>ARCβ</u>

For: 5'-ATGGGCATCTACACGATATTC-3'

Rev: 5'-GCAAGCAAACTAGGATTTCGC-3'

<u>ARCγ</u>

For: 5'-CATACGACCACGAAGCAAGC-3'

Rev: 5'-GACTTGTCCGCTGTATTCATCG-3'

The following parameters were used: 50ºC (30 min); 94ºC (2 min); 40 cycles of 94ºC (15 s), 51ºC (30 s) and 68ºC (3 min); final extension at 68ºC (5 min).

Gene expression analysis

Gene expression of *S.purpuratus* ARCs was assessed by RT-PCR using the SuperScriptII One-Step RT-PCR system with Platinum *Taq* (Invitrogen) with the following gene specific primers:

<u>ARCα</u>

For: 5'-GAATCTCCTCCGCACGTCCCTGTTCTTG-3'

Rev: 5'-ACCATTTAGCAAGAGAGTGGAAACTA-3'

<u>ARCβ</u>

For: 5'-GGGCATCTACACGATATTCCTCTACTTG-3'

Rev: 5'-ACCACTGAGAGACATAGCGAAAACG-3'

<u>ARCγ</u>

For: 5'-ATGCCATACACGGCCTATAT-3'

Rev: 5'-TTACGTATTGTGTAAGACACG-3'

The following parameters were used: 50ºC (30 min); 94ºC (2 min); 40 cycles of 94ºC (30 s), 53ºC (30 s) and 72ºC (2 min); final extension at 72ºC (10 min). PCR amplification products were then separated in a 1% agarose gel. All PCR amplification products were cloned into pCRII-TOPO vector (Invitrogen) and the identity of the resulting RT-PCR product confirmed by DNA sequencing.

Antibody production and purification

Antibodies specific for ARCα, β and γ were raised commercially (anti-ARCα and β by Covalab and ARCγ by Eurogentec) by immunizing rabbits simultaneously with two specific peptides derived from the protein sequences. ARCα peptides: A (DRGAYHLESTFARVC) and B (DFTLPTPNDKVRETC), ARCβ peptides: A (FQLAIPNEMYAETC) and B (CLYLQNFLMNMNFNV) and ARCγ peptides: A (CKAGRVNPTVDLNELRM) and B (FDSNREGGAFHNNNS). Specific antibodies were affinity-purified from immunized rabbit serum using peptides A and B conjugated to Sulfo-Link agarose (Perbio) following the manufacturer's instructions.

Production of recombinant ARC proteins

Rib-hydrolase domains (amino acid residues in brackets) of ARCα (19 to 300), ARC® (23 to 295) and ARCγ (54 to 285) were cloned into pGEX-2TKP and pPICZαA for expression in either bacteria (as GST-fusion proteins) or in yeast (as secreted proteins), respectively.

a) Bacterial expression

GST-fusions of ARC proteins were expressed in *E.coli* strain BL21-Codon Plus (DE3) by induction with 0.1 mM IPTG for 4 h at 18-20°C. The bacterial pellet was resuspended in STE buffer (150 mM NaCl, 10 mM Tris-HCl, 1 mM EDTA, pH 8) containing 5 mM DTT and 100 μg/ml lysozyme, and incubated for 15 min on ice. The bacteria were then lysed with 1.5% Sarkosyl, incubated on ice for 1 h and sonicated. The lysate was clarified by centrifugation at 10,000 *g* for 20 min at 4°C. 2% Triton X-100 was added to the supernatant followed by a further incubation on ice for 30 min. GST-fusion proteins were purified from the lysate by incubation with Glutathione Sepharose 4 Fast Flow (GE Healthcare) and washed with STE buffer containing 1% Triton X-100. After the final wash, sepharose bound proteins were resuspended in Laemmli buffer and analysed by SDS-PAGE.

b) Yeast expression

For yeast expression of ARC proteins all predicted N-glycosylation sites were firstly mutated to Gln residues for ARCα (amino acid residues 31, 137, 153, 169, 249) and to Asp for ARCβ (amino acids 30, 136, 153, 167, 204, 247, 263) using the QuickChange site-directed mutagenesis kit (Statagene). ARC constructs were expressed in *Pichia*

pastoris by induction with 0.5% methanol for 4-7 days as described before [1, 2]. Soluble expressed proteins were concentrated 1/10 using Centriplus concentrators 10,000 MWCO (Amicon) for enzymatic assays.

Preparations of whole eggs and subfractions

a) Live eggs

L. pictus were obtained from Marinus (Long Beach, CA, USA), *S. purpuratus* from Westwind Sealab Supplies (Victoria, BC, Canada).

Eggs were collected in Artificial Sea Water (ASW: 435 mM NaCl, 40 mM $MgCl_2$, 15 mM $MgSO_4$, 11 mM $CaCl_2$, 10 mM KCl, 2.5 mM $NaHCO_3$, 20 mM Tris, 1 mM EDTA, pH 8.0) by intracoelomic injection of *S. purpuratus* with 0.5 M KCl, and subsequently dejellied by filtering through a nylon net filter (Millipore) of 85 μm (for *S. purpuratus*) or 100 μm (for *L. pictus*) and washed in ASW.

b) Stratified eggs

For stratification, eggs were centrifuged on a cushion of 1 M sucrose at 17,500 *g* for 30 min. To dislodge cortical granules [3], eggs were suspended in 400 mM urethane immediately before and during centrifugation. In addition to the distended morphology, stratification was verified by appropriate migration of the nucleus (stained with 10 μg/ml Hoechst 33342), yolk platelets (stained with 1 μM Lysotracker Red DND-99) mitochondria (stained with 300 nM tetramethylrhodamine ethyl ester), and the appearance of the oil droplet (LC Davis and AJ Morgan, unpublished data, 2008).

c) Cortical lawns

Live eggs were washed 3× in Ca^{2+}-free ASW and settled onto poly-L-lysine coated glass coverslips. The eggs were then ruptured with a jet of cold Ca^{2+}-free buffer (0.5 M NaCl, 10 mM KCl, 2.5 mM $NaHCO_3$, 62.5 mM NaOH, 20 mM EGTA, pH 8) which sheared away the cytoplasm leaving the cortices of the eggs bound to the solid support [4].

d) Egg homogenates

Following a previous protocol [5], de-jellied eggs were washed twice in Ca^{2+}-free ASW supplemented with 1 mM EGTA and then twice with Ca^{2+}-free ASW. Finally, eggs were washed with an intracellular medium without EGTA (GluIM: 250 mM potassium gluconate, 250 mM N-methylglucamine, 1 mM $MgCl_2$, 20 mM HEPES, pH 7.2) and homogenized on ice in GluIM (50%) supplemented with an ATP regenerating system (2 mM ATP, 20 U/ml creatine phosphokinase, 20 mM phosphocreatine) and EDTA-free protease inhibitor cocktail (Complete™, Roche) using a pre-chilled Dounce glass tissue homogenizer, size 'A' pestle. Homogenates were centrifuged for 10 s at 13, 000 *g* to remove cortical granules, and the supernatants aliquoted and stored at -80°C.

To examine the effect of EGTA upon vesicle integrity during homogenate preparation (Figure S3), homogenates (50%) were prepared as above with the following modifications: ASW washes and GluIM at all steps was prepared with or without 10 mM EGTA and the removal of cortical granules by centrifugation was omitted.

e) Cell surface complexes (CSCs)

CSCs were prepared as before [6]. Dejellied eggs were washed three times in ASW and suspended in intracellular medium (IM: 220 mM potassium glutamate, 500 mM glycine, 10 mM NaCl, 5 mM $MgCl_2$, 10 mM EGTA, 2.5 mM MgATP, 5 mM dithiothreitol,

EDTA-free protease inhibitor cocktail (Complete™, Roche), pH 6.8). The eggs were washed three times with IM and homogenised in a pre-chilled Dounce glass homogenizer with 3-5 strokes of a tight-fitting pestle. CSCs were pelleted at 1,000 *g* for 1 min and suspended in fresh IM. Centrifugation was repeated until the wash buffer was clear and when only large sheets of egg cortex were visualised under the light microscope. Pellets of CSC were either used immediately or frozen in liquid nitrogen and stored at –80°C.

f) Cortical granules

Cortical granules (CGs) were detached from the CSCs by sucrose displacement [7]: ice-cold 1 M sucrose containing, 1 mM EGTA and EDTA-free protease inhibitor cocktail (Complete™, Roche) was added to a pellet of CSCs by allowing the solution to flow gently down the side of the tube and to wash over the top of the pellet without disturbing it. The sucrose solution was removed and the CSC pellet was resuspended in 10× volume of the sucrose solution. The tube was swirled by hand on ice and incubated on ice for 60 min. CG detachment was checked with phase constrast microscopy. When detachment of CGs was complete, the plasma membrane-vitelline layer (PMVL) components of the CSC were pelleted by centrifugation at 1,000 *g* for 20 min. The supernatant, which contains the CGs was removed and pelleted at 20,000 *g* for 30 min.

g) CSC or CG Lysis

CSCs or CGs were lysed by resuspension in 10 mM HEPES, pH 7 or 10 mM acetate buffer, pH 5 followed by incubation on ice for 10 min. Membranes (particulate fraction) were separated from the soluble fraction by ultracentrifugation at 100,000 *g* for 1 h. The pellets were resuspended in the same volume of lysis buffers. Hypotonic lysis was

confirmed using confocal microscopy and the release of Lysotracker Red staining (AJ Morgan and LC Davis, unpublished data, 2008).

Immunoblotting analysis

Protein samples were mixed with Laemmli sample buffer (under reducing conditions) and proteins resolved in 10% acrylamide gels by SDS-PAGE and blotted onto nitrocellulose membranes using 20 mM sodium phosphate buffer, pH 6.7. Membranes were blocked in PBS (10 mM phosphate buffer, 2.7 mM KCl, 137 mM NaCl, pH 7.4) containing 0.5% Tween and 5% dry skimmed milk and incubated with affinity-purified antibodies. HRP-conjugated anti-rabbit IgG (Sigma) was used as a secondary antibody and specific bands visualized by chemiluminescence using ECL reagents (GE healthcare).

Immunofluorescence analysis

The following primary antibodies were used for immunofluorescence: anti-ARCα (10 to 25 μg/ml), anti-ARCβ (10 to 25 μg/ml), anti-ARCγ (20 μg/ml), anti-hyalin (monoclonal 2B7, [8]), non-immunised rabbit IgG (10 to 25 μg/ml), pre-immune and final bleed sera (1:500). Fluorescently labelled secondary antibodies were $F(ab')_2$ fragments of goat anti-rabbit IgG AlexaFluor 546 (5 μg/ml, Invitrogen), goat anti-rabbit AlexaFluor 488 (1:300, Jackson Immuno) and goat anti-mouse Texas Red (1:300, Invitrogen). In some cases egg preparations were stained with 1 μM Lysotracker Red DND-99 (Invitrogen) for 20 min prior to fixation.

Eggs and cortical lawns were fixed with 4% paraformaldehyde in modified PBS (10 mM phosphate buffer, 10 mM KCl, 450 mM NaCl, pH 7.4). Eight-μm sections of ovary were

fixed with 10% formalin. Fixed samples were permeabilized with 0.2% Triton X-100 where indicated. After blocking non-specific sites with 2% goat serum, samples were incubated with affinity-purified antibodies (or control IgGs) and labelling detected with fluorescently-labelled secondary antibodies. Immunofluorescence was viewed on a confocal laser scanning microscope and images collected in the Multitrack mode which alternates laser lines to reduce bleed through. Excitation/emission (nm): green (488/505-530), red (543/>560).

Immunogold electron microscopy

Eggs were fixed with modified Karnovsky's solution, post-stained with 0.01% OsO_4, and embedded in Spurr's resin. Silver-gold sections (approximately 90 nm) were placed on nickel grids and non-specific sites blocked. Samples were incubated with affinity-purified antibodies (or control IgGs) and labelling detected with secondary antibodies conjugated to 15 nm colloidal gold particles. Sections were post-fixed with 2% glutaraldehyde, and stained with uranyl acetate and lead citrate. Sections were visualized at 100 KeV with a Philips (New York, NY, USA) 410 electron microscope with an Advantage HR CCD camera from Advanced Microscopy Techniques (AMT) run by proprietary software.

Assays for ARC activity

a) TLC assay

The synthesis of [^{32}P]cADPR was assessed by incubation of either yeast-expressed recombinant ARCs or CSC and CG samples with 16.2 nM [^{32}P]-β-NAD$^+$ (GE Healthcare) for 1 h at 20°C and reaction products separated on silica gel thin layer chromatography (TLC) sheets (Merck) in a mixture of 30% water / 70% ethanol / 0.2 M

ammonium bicarbonate [9]. Autoradiography was carried out after exposure on a storage phosphor screen (Typhoon, GE Healthcare). Spots were verified by the co-migration position of authentic β-NAD^+, cADPR and ADPR. Synthesis of cADPR was also independently confirmed by a cycling assay which measures cADPR mass [10]. Specific ARC activity of cortical granules was calculated by dividing the absolute cADPR production by the density of the ARCβ band on an immunoblot.

b) Ca^{2+} release assay

L. pictus egg homogenates (with cortical granules removed) were sequentially diluted from 50% stocks to 2.5% over a period of 3 h at 17°C using GluIM, containing an ATP regenerating system and 3 μM Fluo-3 (Invitrogen) [5]. Fluorescence was monitored at 17°C in a PerkinElmer LS-50B fluorimeter using excitation 506 ± 4 nm, and emission 526 ± 4 nm. To assess whether transport inhibitors affected either intracellular Ca^{2+} release channels or ARC activity, messengers or β-NAD^+ respectively were added to the homogenate following a 3 minute pre-incubation with either 0.1% DMSO (control) or the indicated concentration of inhibitor. Agents were added from 100× stocks. Fluorescence was calibrated using the standard equation $[Ca^{2+}] = K_d (F - F_{min})/(F_{max} - F)$, using a K_d of 0.4 μM; F_{min} (where F is fluorescence) and F_{max} were determined by the addition of 500 μM EGTA and 10 mM Ca^{2+} respectively at the end of each run.

Nucleotide uptake

CSCs were incubated for various times with ~16 nM [^{32}P]β-NAD^+ in incubation buffer (GluIM supplemented with 10 mM EGTA and 100 μM ATP-Na^+ salt) at 20°C and separated from free nucleotide using vacuum filtration (Whatman GF/B filters) and three

washes of ice-cold GluIM supplemented with 10 mM EGTA and 160 μM unlabelled β-NAD$^+$. For zero time points CSCs were added to excess ice-cold wash buffer containing [^{32}P]β-NAD$^+$ and filtered immediately. Non-specific binding was determined by preincubation with 100 μM unlabelled 'cold' β-NAD$^+$. Luminal ^{32}P was distinguished from ^{32}P bound to the surface by including 300 μM digitonin in the wash buffer once the CSCs were filtered. Digitonin immediately lyses cortical granules without grossly affecting their morphology (AJ Morgan unpublished data, 2008). Where indicated, dipyridamole (DPM), NBTI, or Indoprofen were preincubated for 2 min prior to addition of [^{32}P]β-NAD$^+$. GF/B filters were exposed to a phosphor-storage screen and scanned on a Typhoon imager (GE Healthcare).

In order to identify the ^{32}P-labelled nucleotides that accumulate in the cortical granule lumen, CSCs were preincubated for 2 min with DMSO or DPM prior to incubation for 18 min with 50 nM [^{32}P]β-NAD$^+$ in incubation buffer (GluIM supplemented with 10 mM EGTA and 100 μM ATP-Na$^+$ salt) at 20°C. Following rapid vacuum filtration (Whatman GF/B filters) and three washes of ice-cold GluIM supplemented with 10 mM EGTA and 500 μM unlabelled β-NAD$^+$, the cortical granules were lysed in 4.8% TCA to release ^{32}P -labelled luminal nucleotides, precipitate protein and terminate ARC activity. Precipitated protein was pelleted and the supernatants were concentrated 10-fold under vacuum before separation and identification of ^{32}P-labelled nucleotides by TLC analysis (see TLC assay above). Time zero intensities were subtracted from each nucleotide spot and data normalized to the respective DMSO controls.

Monitoring intracellular Ca^{2+} concentration and vital staining in intact eggs

L. pictus eggs on poly-lysine-coated glass coverslips were maintained in ASW at room temperature and microinjected with a Ca^{2+}-sensitive fluorescent dye, fluo-4 dextran (Invitrogen; pipette concentration 1 mM in 250 mM KCl, Tris, pH 8). Eggs were then imaged by confocal laser scanning microscopy using a 40 × objective (excitation 488 nm, emission >505 nm). In fertilization experiments (using 0.1% ejaculate), the following agents were added for different times (in parentheses): dipyridamole (3 min), nitrobenzylthioinosine (45-90 min), Indoprofen (5 min), isobutylmethylxanthine (5 min) or indomethacin (5 min). Control eggs were incubated with the appropriate vehicle concentration (0.03-0.5% DMSO). Images are self-ratioed against a pre-stimulation image (F/F_0), depicted on a common ratio scale (0.95 - 5.00) and pseudo-coloured with a Rainbow look-up table. Wave kinetics were calculated as the time to cross the egg (from the initiation site to the antipode), with the transit times recorded when the spike height was 50% of its maximum.

To examine Ca^{2+} release in response to nucleotides, fluo-4-dextran-loaded eggs were microinjected using a second micropipette loaded with nucleotide plus a fluorescent injection marker, Alexa Fluor 647 Dextran (50 μM in the pipette; excitation 633 nm, emission >650 nm). Pipette concentrations: β-NAD^+ (5-10 mM, plus 100 μM EGTA), cADPR (30 μM). No Ca^{2+} response was observed when these nucleotides were absent (Figure 4 Legend, and see [11]). Nucleotide $[Ca^{2+}]_i$ responses were normalised to the ΔF of control eggs.

To estimate the *intracellular* dipyridamole concentration, we exploited the intrinsic, weak UV fluorescence of dipyridamole recorded on the confocal microscope (excitation 364

nm, emission >385 nm). Acquisition settings were such that unlabelled eggs showed little autofluorescence, but the signal increased upon addition of 30 μM dipyridamole (extracellular concentration) to the bath.

The endoplasmic reticulum was stained in live eggs according to previous methods [12]. In summary, a saturated solution of DiI ($DiIC_{18}$ (3), Invitrogen) was prepared in soyabean oil (Sigma-Aldrich) and microinjected into live eggs (injection pressure ~5500 hPa for 6-8 s). The DiI diffused from the central oil droplet into the contiguous membrane system of the ER in 15-30 min and was imaged on a confocal laser scanning microscope using a 40 × objective (1-μm slice) and a red filter set (excitation/emission: 543/>560 nm). For colocalization of ER and acidic vesicles, eggs were incubated with 1 μM Lysotracker Green DND-26 (Invitrogen) immediately after DiI injection. Following dye equilibration, red and green channels were collected in the confocal Multitrack mode (to reduce potential bleed-through) and using a 63 × objective (0.7-μm slice).

Data Analysis

Data are presented as the mean ± standard error of the mean of n preparations. Statistical analysis was conducted using Student's t test (for two means) and an analysis of variance (ANOVA) followed by a Tukey or Dunnett post test (for multiple means) and significance assumed at $P<0.05$.

SUPPLEMENTAL FIGURES

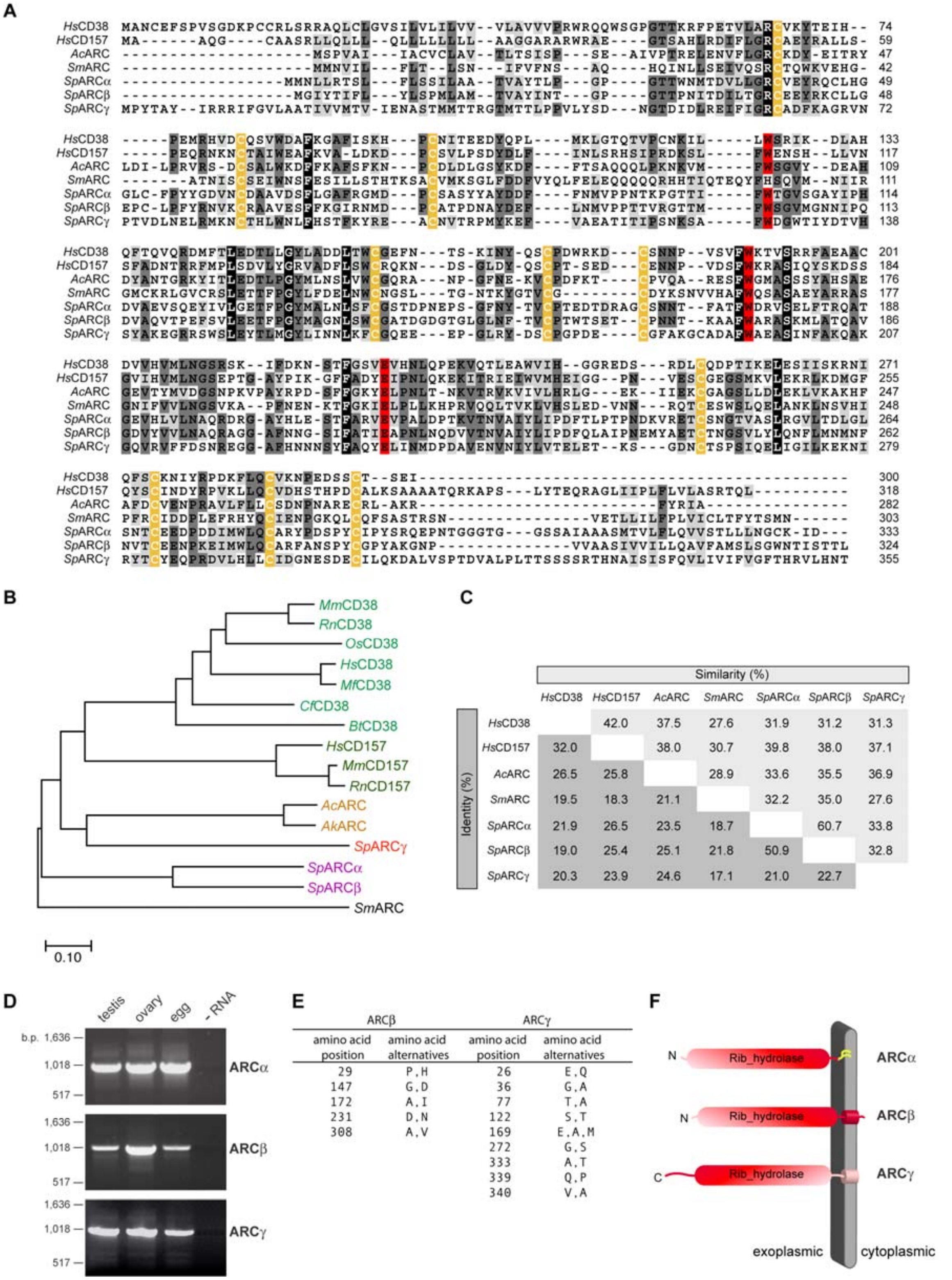

Figure S1 Comparative analysis of *S. purpuratus* ARCs with other ARC family members.

(A) Sequence alignment of ARC family proteins by ClustalW. Catalytic residues (red), conserved cysteines (yellow), all other conserved residues (black), identical residues (dark grey) and similar residues (light grey). Numbers on the right denote the last amino acid residue for each line. (B) Phylogenetic relationship of cloned ARC proteins across different species using the Neighbor-Joining method by MEGA3 software. The horizontal bar shows the number of amino acid substitutions per site. (C) Percentage of identical (dark grey) and similar (light grey) residues amongst ARC proteins across species. Values relate to the alignment in (A). (D) Expression of ARCα, ARCβ and ARCγ was detected by RT-PCR using *S. purpuratus* ovary, testis and egg total RNA and resolved in 1% agarose-TAE ethidium bromide gel. (E) Comparison of nucleotide sequences corresponding to ARCβ and ARCγ clones obtained from independent RT-PCR reactions revealed a high number of polymorphisms, as shown previously for sea urchin genes [13, 14]. Tabulated are the amino acid alternatives found in multiple independent clones. Next to each amino acid position, the observed alternative amino acids are indicated in one-letter code. Polymorphisms at amino acids 29 and 308 (ARCβ) and 36 and 77 (ARCγ) explain some of the differences between our sequences and those found by [15]. (F) Schematic representation of predicted topology of mature *S. purpuratus* ARC proteins. The domains indicated are ribosyl-hydrolase (Rib-hydrolase) predicted by Pfam (accession number PF02267), GPI-anchor (yellow lines) predicted by GPI-SOM and transmembrane segments (cylinders) predicted by TMHMM2. Signal peptides and signal anchors present in the precursor proteins were predicted by SignalP. ARCα has a predicted cleavable signal peptide attached via a GPI-anchor like CD157 [16] and the Schistosoma ARC [17]; ARCβ also has a predicted cleavable signal peptide, bound through a transmembrane segment close to the C-terminal tail that extends into the cytoplasm; ARCγ has a predicted signal anchor, transmembrane sequence at the N terminus with an exoplasmic C terminus, in a similar manner to CD38 [16]

Species abbreviations: *Ac* (*Aplysia californica*), *Ak* (*Aplysia kurodai*), *Bt* (*Bos taurus*), *Cf* (*Canis familiaris*), *Hs* (*Homo sapiens*), *Mf* (*Macaca fascicularis*), *Mm* (*Mus musculus*), *Oc* (*Oryctolagus cuniculus*), *Rn* (*Rattus norvegicus*), *Sm* (*Schistosoma mansoni*) and *Sp* (*Strongylocentrotus purpuratus*). Protein accession numbers: *Ac*ARC (P29241), *Ak*ARC (BAA07537), *Bt*CD38 (AAI26709), *Cf*CD38 (NP_001003143), *Hs*CD38 (NP_001766),

*Hs*CD157 (NP_004325), *Mf*CD38(AAT36330), *Mm*CD38 (NP_031672), *Mm*CD157 (NP_033893), *Oc*CD38 (JC7323), *Rn*CD38 (NP_037259), *Rn*CD157 (Q63072), *Sm*ARC (AAX35328), *Sp*ARCα (EF544483), *Sp*ARCβ (EF544484) and *Sp*ARCγ (EF544485).

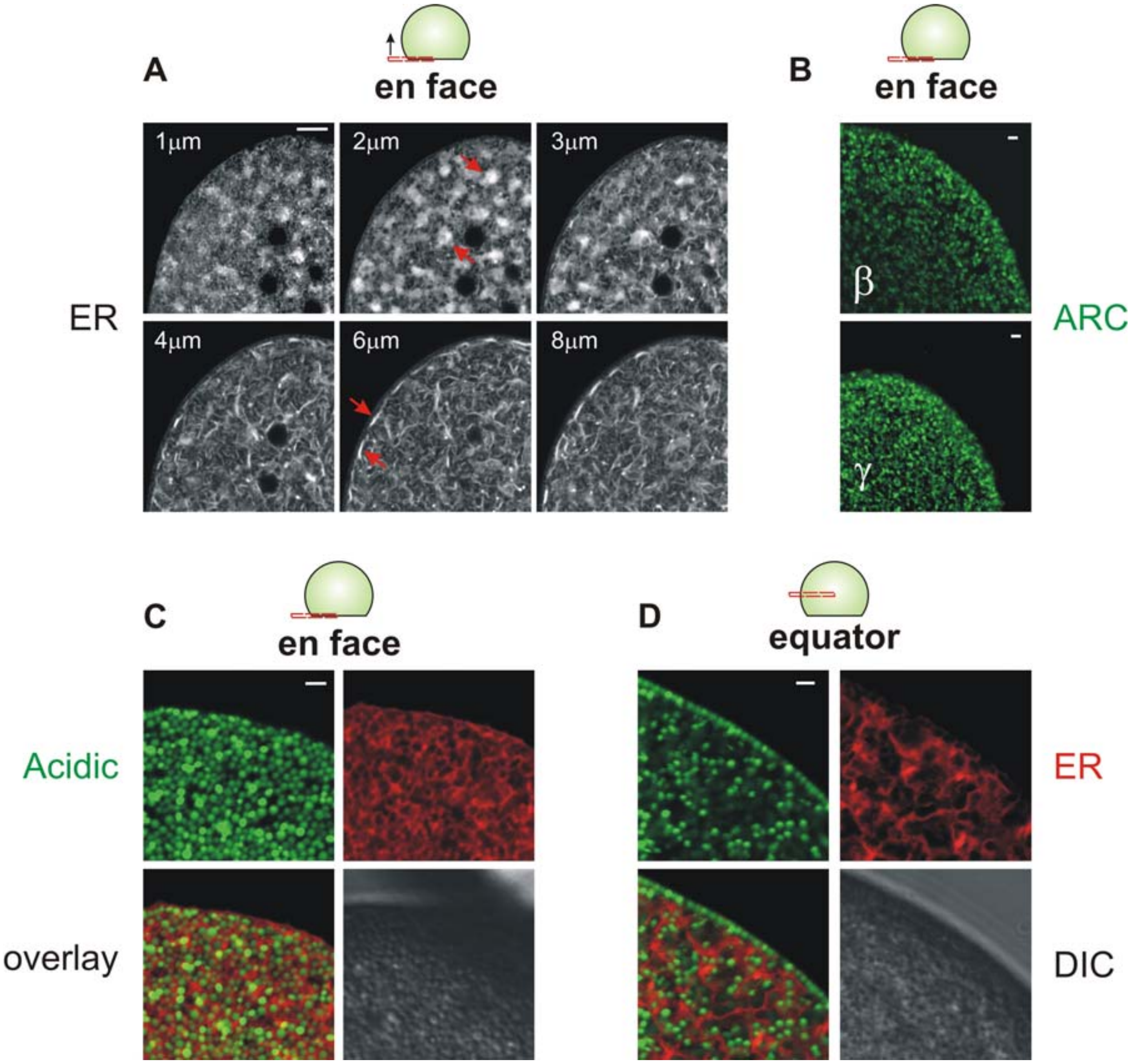

Figure S2 Comparison of labelling endoplasmic reticulum (ER), ARCs, and acidic vesicles

Endoplasmic reticulum (A,C,D) was labelled with 'DiI' [12], and acidic vesicles (C,D) with 1 μM Lysotracker Green DND-26 (LTG). Images were collected on a confocal laser scanning microscope using standard green (LTG) and red (DiI) filters. (A) Serial confocal sections of a live egg stained with DiI only where 1-8 μm are labels referring to the distance from (and including) the first *en face* section, and thence upwards. The tubular reticulum at the peripheral cortex (1 μm), the sub-cortical cisternae (2 μm) and deeper membrane sheets (> 3 μm) are consistent with previous observations [12]. Note that even

the prominent cisternae are too deep, too flat and too large for ARC-containing structures (diameter of 5-6 µm, see *red* arrows: *top view* in the 2-µm section, *side view* in the 6-µm section). (B) Fixed eggs were stained for ARCβ or ARCγ as detailed in Materials and Methods and viewed *en face*. (C,D) Co-staining of acidic vesicles with LTG (‘Acidic”) and ER in the same, live egg (DIC, differential interference contrast). (C) An *en face* section shows a vesicular LTG pattern whereas the cortical ER is a fine reticulum without cisternae. (D) A deeper, equatorial slice shows a peripheral arc of cortical granules labelled with LTG and the inner, flanking ER tubules. Scale bars represent 10 µm (A) and 2 µm (B-D).

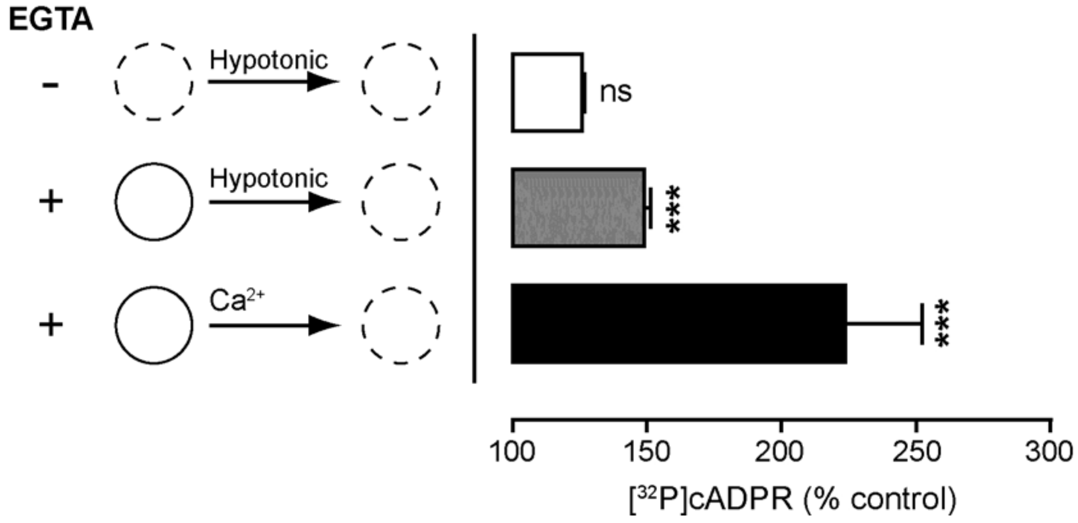

Figure S3 EGTA preserves vesicular integrity in egg homogenates

L. pictus egg homogenates containing cortical granules were prepared in the presence (+) or absence (-) of 10 mM EGTA, pH 7.2 using a modification of previous methods [5]. Egg homogenates (5%) were assayed for [^{32}P]cADPR production from ~16 nM [^{32}P]β-NAD^{+} in 1 h (final EGTA concentration of 1 mM in the enzyme assay for all three conditions). Radiolabelled products were analysed by TLC and the cADPR component of the total radioactivity expressed as a percentage of the corresponding intact homogenate (% control). Spot intensities (a.u.), *Intact (control)*: –EGTA, 420 ± 6; Hypotonic lysis, 1120 ± 30; Ca^{2+} lysis, 1730 ± 200. Egg homogenates in the presence of EGTA have increased cADPR production upon hypotonic lysis (in 10 mM HEPES, pH 7.0) or by addition of 1 mM Ca^{2+} (free [Ca^{2+}] ~ 20 µM). pH was maintained at pH 7.0 upon addition of Ca^{2+} to EGTA by co-addition of KOH. Although not optimal for ARC activity, pH 7.0 is optimal for Ca^{2+} chelation by EGTA. $P<0.001$*** (lysis versus intact); for (+) EGTA, lysis and Ca^{2+} were not significantly different from each other ($P>0.05$). Data are expressed as the mean ± S.E.M. (n = 3).

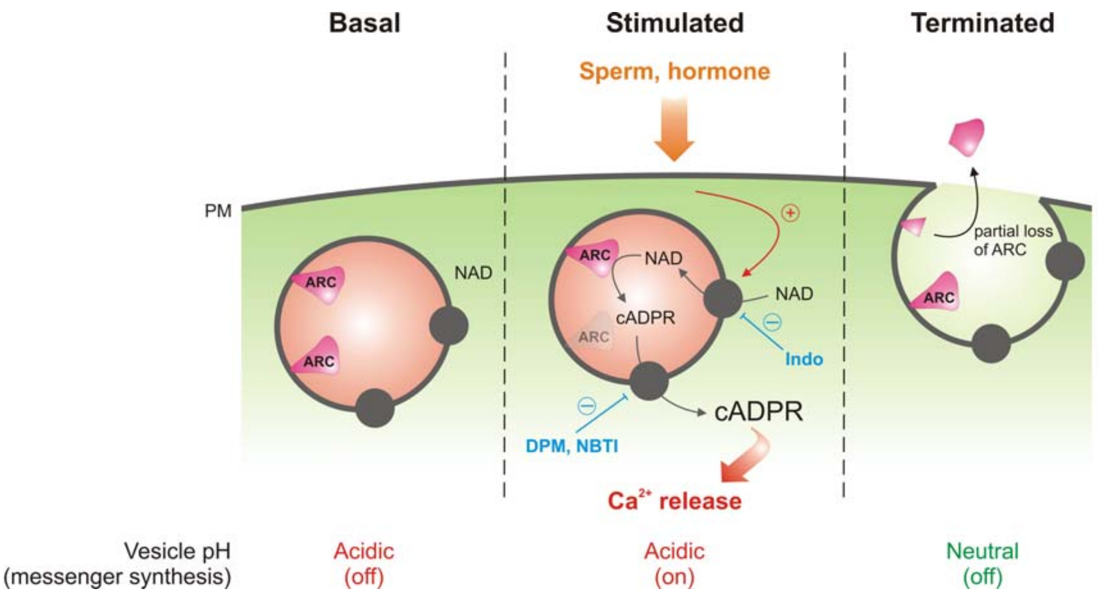

Figure S4 Model of receptor coupling mechanisms to ARC

In the unstimulated (basal) cell, ARC is sequestered in the lumen of an acidic organelle and is effectively inactive (e.g. limited by substrate availability). Extracellular stimuli trigger nucleotide transport across the vesicle membrane and cADPR synthesized within the lumen is translocated to the cytoplasmic ryanodine receptors to evoke Ca^{2+} release (the lower ARC symbol is 'greyed out' for clarity). ARC activity may be terminated by multiple mechanisms: fusion of the exocytotic vesicle with the plasma membrane (PM) elevates the luminal pH to inhibitory levels; exocytosis dilutes substrate/product, extrudes some ARCβ from the cell (perhaps in concert with proteolytic clipping); nucleotide transport may also be inhibited. It is unlikely that β-NAD^+ transporters and ARC are simultaneously, fully active under basal conditions since this would deleteriously consume cytosolic pools of β-NAD^+. Our model does not differentiate between regulation at the level of the β-NAD^+ or cADPR transport.

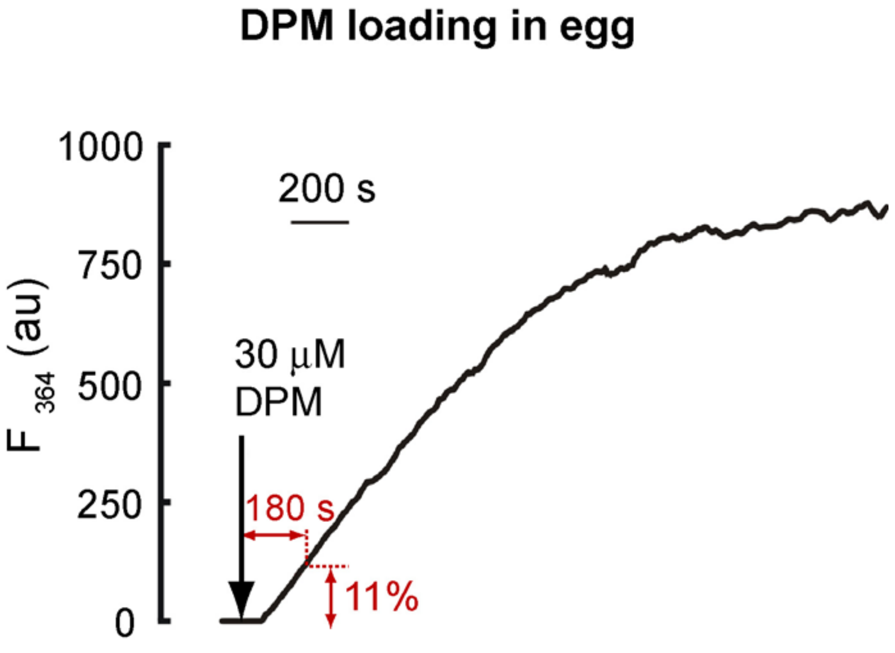

Figure S5 Dipyridamole loading of intact eggs

The weak UV fluorescence of dipyridamole was exploited as an index of its time course of loading into intact eggs. Unlabeled eggs were mounted on a confocal microscope equipped with a UV laser and 30 μM dipyridamole (DPM) added to the extracellular medium at the time indicated and the intracellular fluorescence recorded until a plateau was attained. At equilibrium, the intracellular concentration of DPM is, at most, the same as the extracellular concentration. A 3-min preincubation (*cf.* Fig. 4) gives an upper estimate for the intracellular DPM concentration as 11.3 ± 1.8% of the bath concentration.

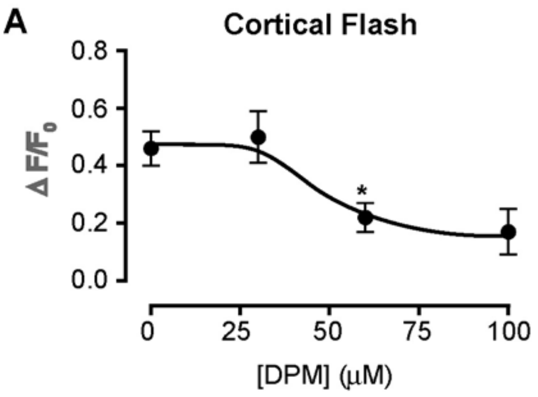

Figure S6 Effect of dipyridamole upon fertilization-induced Ca^{2+} responses

Data are derived from the experiments performed in Figure 4A, B. (A) Effect of DPM upon the cortical flash amplitude. (B) Effect of DPM upon the rise time of the main Ca^{2+} response measured at the wave initiation site (the point of sperm entry, green square on schematic Figure 4A), and the antipode (red square). Longer times reflect slower rates of rise of Ca^{2+}. * $P<0.05$, ** $P<0.01$ compared to 0 μM DPM.

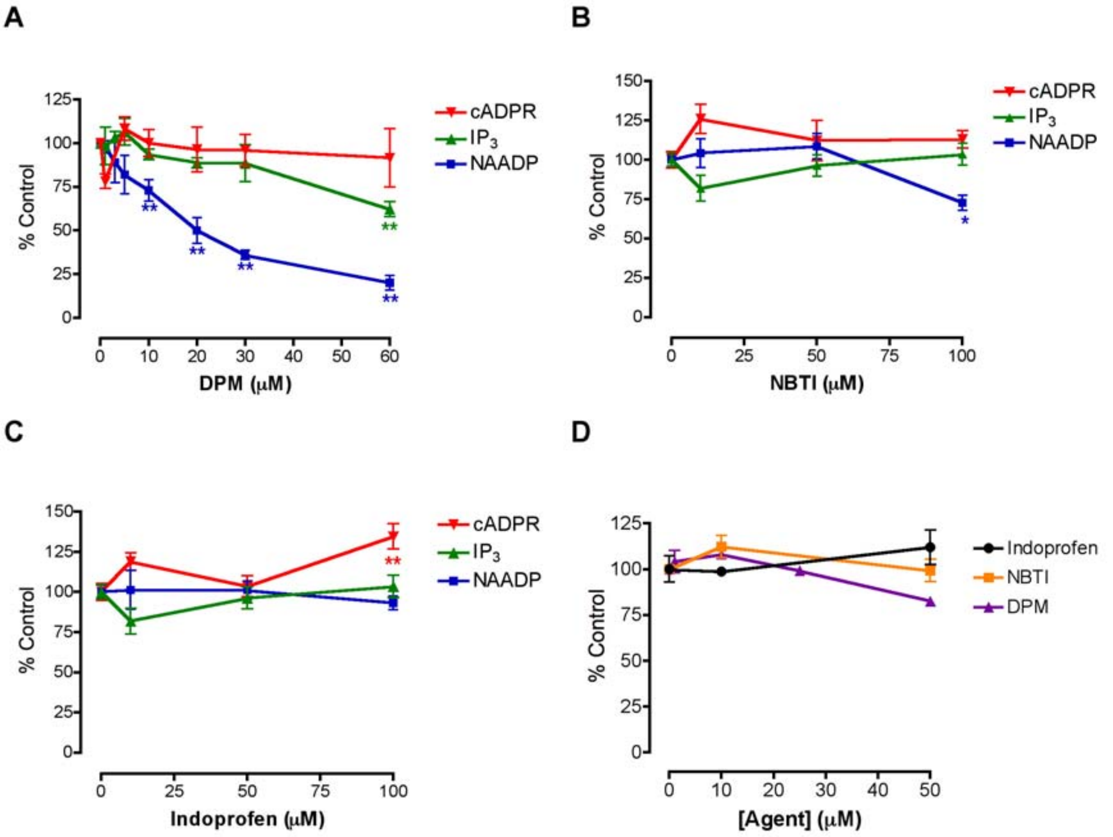

Figure S7 Dipyridamole, NBTI and Indoprofen effects upon ARC activity and Ca^{2+} release

(A-C) Effect of transport inhibitors upon Ca^{2+} release in sea urchin egg homogenate measured with fluo-3. Intracellular channels were stimulated by addition of 0.5 μM IP_3, 30 nM cADPR or 50 nM NAADP and the amplitude of their Ca^{2+} responses measured in the absence or presence of various concentrations of DPM (A), NBTI (B) or Indoprofen (C). Inhibitors (or DMSO vehicle) were added to the cuvette 3 min prior to second messenger addition. Data were calibrated as the $\Delta[Ca^{2+}]$ released (nM) and expressed as a percentage of the control ± SEM (n = 3-6). $\Delta[Ca^{2+}]$ (nM) for DMSO controls: (A) IP_3, 50 ± 5; cADPR: 88 ± 6; NAADP: 93 ± 1. (B,C) IP_3: 75 ± 7; cADPR: 69 ± 5; NAADP: 108 ± 5. (D) Effect of DPM, NBTI and Indoprofen upon ARC activity in egg homogenates assessed by conversion of 100 μM β-NAD^+ to cADPR. $\Delta[Ca^{2+}]$ (nM) for DMSO controls: DPM: 76 ± 14; NBTI, Indo: 99 ± 9. Significant inhibition by agent compared to

the respective control (**, P<0.01, *, P<0.1), else the agent had no significant effect upon responses (P>0.05). Given the maximal intracellular concentration attained (Figure S5), the extracellular DPM concentrations used in intact eggs would not affect cADPR- and IP_3-induced Ca^{2+} release, whereas NAADP-induced Ca^{2+} release would only be directly affected at 100 μM DPM in the bath.

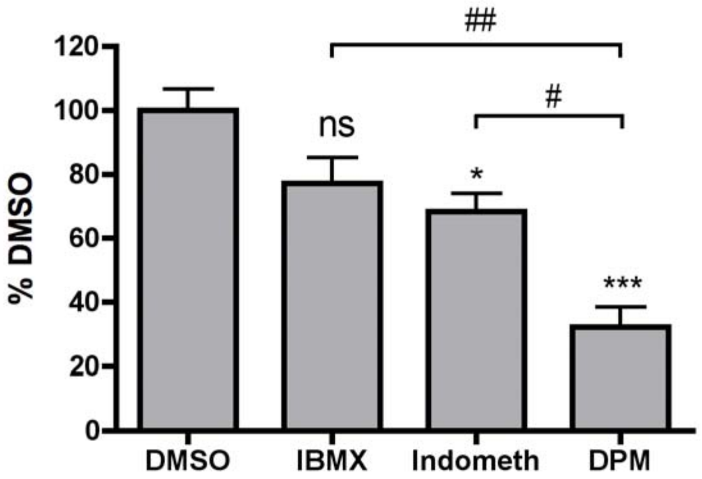

Figure S8 Comparison of the effect of inhibitors of phosphodiesterase, cyclooxygenase and nucleotide transport upon fertilization-induced Ca^{2+} responses. DPM can also inhibit phosphodiesterases (PDE) [18], and indoprofen is also a cyclo-oxygenase inhibitor [19] so the effects of transport inhibition with DPM were compared with the PDE inhibitor isobutylmethylxanthine (IBMX) [20] and the cyclo-oxygenase inhibitor, indomethacin at maximal concentrations [19]. Experiments were all performed in heparin-injected eggs since they exhibit greatest sensitivity to transport inhibition (Figure 4F). Heparinized eggs were pre-treated with 0.1-0.2% DMSO, 1 mM IBMX or 100 μM indomethacin for 5 min, or with 60 μM DPM for 3 min (n = 11-37 eggs). Data represent the amplitude of the global main Ca^{2+} peak following fertilization, normalized to the DMSO control response ($\Delta F/F_0 = 1.78 \pm 0.16$, n = 37). Compared to DMSO control: ns, not significant, * $P < 0.05$, *** $P < 0.001$. Compared to DPM: # $P < 0.05$, ## $P < 0.01$. Neither IBMX nor indomethacin mimicked DPM, and the small effect of indomethacin is likely due to its own ability to inhibit nucleotide transport at high concentrations [21].

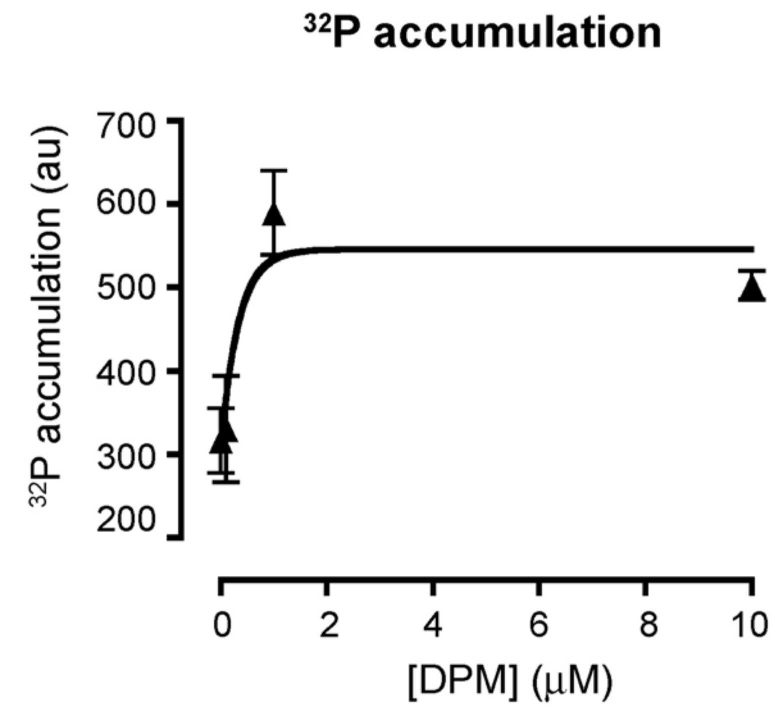

Figure S9 Effect of Dipyridamole on [^{32}P]β-NAD^{+} uptake into cortical granules

DPM dose-dependently enhances the accumulation of ^{32}P in CSCs incubated with 16 nM [^{32}P]β-NAD^{+} for 5 min. Au, arbitrary units. All data are expressed as the mean ± s.e.m. (*n* = 3).

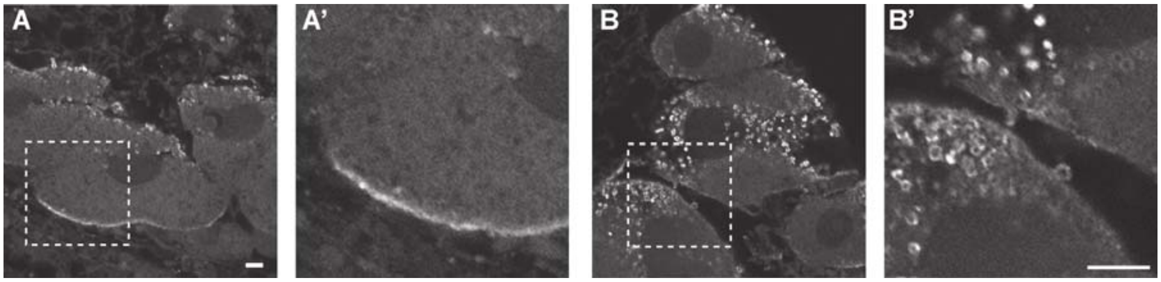

Figure S10 ARCα staining is vesicular in ovary sections

S. purpuratus ovary sections at different stages of maturation were stained with anti-ARCα. ARCα staining was present at the plasma membrane (A, A') later on in oocyte maturation, whilst at an earlier stage ARCα is located to intracellular vesicles (B, B'). Scale bars represent 10 μm.

SUPPLEMENTAL REFERENCES

1. Munshi, C., Aarhus, R., Graeff, R., Walseth, T.F., Levitt, D., and Lee, H.C. (2000). Identification of the enzymatic active site of CD38 by site-directed mutagenesis. J. Biol. Chem. *275*, 21566-21571.
2. Munshi, C., Thiel, D.J., Mathews, II, Aarhus, R., Walseth, T.F., and Lee, H.C. (1999). Characterization of the active site of ADP-ribosyl cyclase. J. Biol. Chem. *274*, 30770-30777.
3. Hylander, B.L., and Summers, R.G. (1981). The effect of local anesthetics and ammonia on cortical granule-plasma membrane attachment in the sea urchin egg. Dev. Biol. *86*, 1-11.
4. Vacquier, V.D. (1975). The isolation of intact cortical granules from sea urchin eggs: calcium lons trigger granule discharge. Dev. Biol. *43*, 62-74.
5. Morgan, A.J., Churchill, G.C., Masgrau, R., Ruas, M., Davis, L.C., Billington, R.A., Patel, S., Yamasaki, M., Thomas, J.M., Genazzani, A.A., et al. (2006). Methods in cADPR and NAADP research. In Methods in Calcium Signalling, J.W. Putney, Jr., ed. (Boca Raton: CRC Press), pp. 265-334.
6. Whalley, T., Grossmann, J.G., and Wess, T.J. (2000). An analysis of the lamellar structure of sea urchin egg cortical granules using X-ray scattering. FEBS Lett *482*, 242-246.
7. Kinsey, W.H., Decker, G.L., and Lennarz, W.J. (1980). Isolation and partial characterization of the plasma membrane of the sea urchin egg. J. Cell. Biol. *87*, 248-254.
8. Wessel, G.M., Berg, L., Adelson, D.L., Cannon, G., and McClay, D.R. (1998). A molecular analysis of hyalin--a substrate for cell adhesion in the hyaline layer of the sea urchin embryo. Dev. Biol. *193*, 115-126.
9. Higashida, H., Yokoyama, S., Hashii, M., Taketo, M., Higashida, M., Takayasu, T., Ohshima, T., Takasawa, S., Okamoto, H., and Noda, M. (1997). Muscarinic receptor-mediated dual regulation of ADP-ribosyl cyclase in NG108-15 neuronal cell membranes. J. Biol. Chem. *272*, 31272-31277.
10. Graeff, R., and Lee, H.C. (2002). A novel cycling assay for cellular cADP-ribose with nanomolar sensitivity. Biochem. J. *361*, 379-384.
11. Morgan, A.J., and Galione, A. (2007). Fertilization and nicotinic acid adenine dinucleotide phosphate induce pH changes in acidic Ca^{2+} stores in sea urchin eggs. J. Biol. Chem. *282*, 37730-37737.
12. Terasaki, M., and Jaffe, L.A. (1991). Organization of the sea urchin egg endoplasmic reticulum and its reorganization at fertilization. J. Cell Biol. *114*, 929-940.
13. Sodergren, E., Weinstock, G.M., Davidson, E.H., Cameron, R.A., Gibbs, R.A., Angerer, R.C., Angerer, L.M., Arnone, M.I., Burgess, D.R., Burke, R.D., et al. (2006). The genome of the sea urchin *Strongylocentrotus purpuratus*. Science *314*, 941-952.
14. Britten, R.J., Cetta, A., and Davidson, E.H. (1978). The single-copy DNA sequence polymorphism of the sea urchin *Strongylocentrotus purpuratus*. Cell *15*, 1175-1186.

15. Churamani, D., Boulware, M.J., Geach, T.J., Martin, A.C., Moy, G.W., Su, Y.H., Vacquier, V.D., Marchant, J.S., Dale, L., and Patel, S. (2007). Molecular characterization of a novel intracellular ADP-ribosyl cyclase. PLoS ONE *2*, e797.
16. Lee, H.C. (2006). Structure and enzymatic functions of human CD38. Molecular Medicine (Cambridge, Mass.) *12*, 317-323.
17. Goodrich, S.P., Muller-Steffner, H., Osman, A., Moutin, M.J., Kusser, K., Roberts, A., Woodland, D.L., Randall, T.D., Kellenberger, E., Loverde, P.T., et al. (2005). Production of calcium-mobilizing metabolites by a novel member of the ADP-ribosyl cyclase family expressed in *Schistosoma mansoni*. Biochemistry *44*, 11082-11097.
18. Kim, H.H., and Liao, J.K. (2008). Translational therapeutics of dipyridamole. Arteriosclerosis, Thrombosis, and Vascular Biology *28*, s39-42.
19. Reid, G., Wielinga, P., Zelcer, N., van der Heijden, I., Kuil, A., de Haas, M., Wijnholds, J., and Borst, P. (2003). The human multidrug resistance protein MRP4 functions as a prostaglandin efflux transporter and is inhibited by nonsteroidal antiinflammatory drugs. Proc. Natl. Acad. Sci. USA *100*, 9244-9249.
20. Morgan, A.J., Murray, K.J., and Challiss, R.A. (1993). Comparison of the effect of isobutylmethylxanthine and phosphodiesterase-selective inhibitors on cAMP levels in SH-SY5Y neuroblastoma cells. Biochem. Pharmacol. *45*, 2373-2380.
21. Jedlitschky, G., Tirschmann, K., Lubenow, L.E., Nieuwenhuis, H.K., Akkerman, J.W., Greinacher, A., and Kroemer, H.K. (2004). The nucleotide transporter MRP4 (ABCC4) is highly expressed in human platelets and present in dense granules, indicating a role in mediator storage. Blood *104*, 3603-3610.
